# Rapid repetitive syllable sounds associate with episodic memory, executive function, and working memory in cognitively healthy and subjectively impaired older adults

**DOI:** 10.1007/s11357-025-01739-x

**Published:** 2025-06-24

**Authors:** Julia Giffard, Renjie Li, Eddy Roccati, Adam P. Vogel, Aidan D. Bindoff, Lynette R. Goldberg, Quan Bai, Jane Alty

**Affiliations:** 1 Wicking Dementia Research and Education Centre, College of Health and Medicine, Medical Science Precinct, Medical Sciences 1, University of Tasmania, Level 4, 17 Liverpool St, Hobart, TAS 7000 Australia; 2 Audiology and Speech Pathology, The University of Melbourne, 550 Swanston St, Parkville, VIC 3010 Australia; 3 Redenlab Inc. Australia, 585 Little Collins St, Suite 669, Melbourne, VIC 3000 Australia; 4 School of Information and Communication Technology, Centenary Building, University of Tasmania, Grosvenor St, Sandy Bay, TAS 7005 Australia; 5 Tasmanian School of Medicine, Medical Science 1, University of Tasmania, Level 1, 17 Liverpool St, Hobart, TAS 7000 Australia; 6 Neurology Department, Royal Hobart Hospital, 48 Liverpool St, Hobart, TAS 7000 Australia

**Keywords:** Alzheimer’s disease, Subjective cognitive impairment, Diadochokinesis, Executive function, Episodic memory, Working memory

## Abstract

Non-invasive screening tools for Alzheimer’s disease (AD) risk are needed. Decline in episodic memory and subjective cognitive impairment (SCI) are both associated with elevated AD risk. We investigated associations between three cognitive domains (episodic memory, executive function, and working memory) and motor speech performance in older adults with healthy cognition (HC) or SCI. In total, 1014 community-dwelling participants (cross-sectional sample: 843 HC, mean 66.9 years, 72.8% female; 171 SCI, mean 66.3 years, 70.8% female) remotely completed cognitive tests (Paired Associates Learning and Spatial Working Memory from the Cambridge Neuropsychological Test Automated Battery) and motor speech tests (Tasmanian [TAS] Test). We evaluated whether acoustic motor speech features extracted from 10-s tests of “pa,” “ta,” or “ka” syllable repetition improved episodic memory, executive function, and working memory regression models over age, sex, education, anxiety, and depression (ΔAIC > 2). Motor speech features improved estimation of cognitive scores over fixed demographic and clinical variables in each group and cognitive domain. In summary, we found associations between motor speech performance and cognition in older adults. Short motor speech tests demonstrate potential for non-invasive, cost-effective AD risk screening. Longitudinal research is needed to investigate relationships between individual motor speech features and cognitive changes confirmed to result from the progression of AD.

**Supplementary Information:**

The online version contains supplementary material available at 10.1007/s11357-025-01739-x.

## Introduction

Alzheimer’s disease (AD) is the most common cause of dementia, accounting for up to 70% of cases [1]. AD is a complex neurodegenerative condition characterized by the accumulation of amyloid beta plaques and tau proteins in the brain followed by cognitive decline [2]. AD symptomatology progresses over 15–25 years, from healthy cognition (HC) with underlying covert pathology, through mild cognitive impairment (MCI), which may be preceded by subjective cognitive impairment (SCI), to dementia [3]. Fourteen modifiable risk factors, such as hypertension and smoking, have been determined to account for up to 45% of dementia cases [4]. Early intervention options are increasing with the development of new disease-modifying therapies [5, 6].

Accessible, cost-effective, and non-invasive methods are needed to detect AD during its preclinical HC and SCI stages to facilitate early risk modification and treatment [2, 7, 8]. Current early detection techniques and assessments include blood- and CSF-based biomarkers, neuropsychological assessments, and PET brain imaging. These methods, however, are not scalable to the population level due to dependencies including specialist equipment, skilled administration, and trained interpretation. Cognitive assessments such as the Montreal Cognitive Assessment (MoCA) may be more scalable but lack sensitivity to earlier preclinical and SCI stages, which are characterized by a lack of objective cognitive impairment.

Motor function tests show promise for early detection of AD [9]. Gait deterioration begins in the preclinical stage about 10 years before dementia diagnosis, leading to slower gait and greater stride variability in people with dementia compared to healthy older adults [10, 11]. Interest is growing in other motor tests such as finger tapping (FT) which is an established method of assessing upper limb motor function but has been used in few studies of cognitive decline and discrimination of preclinical AD, MCI, and dementia [12, 13]. Since AD pathology typically begins in the hippocampus, a known flag of elevated risk of covert AD is subtle decline in episodic memory [14–17]. FT shows links to cognitive performance, including the domain of episodic memory [13, 18].

Motor function is also a crucial component of speech production. Difficulties with sequencing and repetition are known to occur in people with dementia [19–21]. Diadochokinetic (DDK) motor speech tests involve participants rapidly repeating syllables such as “pa", “ta", or “ka” individually (as in “papapa”), or sequentially (as in “pataka”) [22]. In younger adults, performance on DDK tests is associated with executive function, specifically cognitive switching [23, 24]. Higher cognitive switching and updating of working memory are associated with higher accuracy on sequential DDK tests, and higher cognitive switching is associated with slower rates on monosyllabic and sequential DDK tests [24].

DDK tests may complement FT- and gait-based assessments of cognition by adding a focus on the functional daily task of speaking. DDK tests do not require specialist equipment such as gait mats, and they remain accessible for people with reduced mobility, ambulation, and balance. We drew upon the findings in DDK and cognition in younger adults [23, 24], and the emerging evidence in older adults of FT tests aiding the estimation of episodic memory, executive function, and working memory [13, 18, 25], to investigate whether fast, monosyllabic DDK tests elicited similar associations in older adults.

Focusing first on episodic memory due to its sensitivity to AD [14–17], we hypothesized that performance features derived from three monosyllabic DDK tests (“pa", “ta", and “ka”) would improve estimation of episodic memory scores in a cognitively healthy group of older adults over null models accounting for readily obtainable demographic and clinical details (age, sex, education, anxiety, and depression). Second, we hypothesized that features from each DDK test would, likewise, improve model fit for executive function and working memory scores.

Since SCI itself is a risk factor for dementia (an estimated 2.3% of affected older adults progress to dementia annually; double the rate of those without SCI) [26], we undertook a secondary analysis in participants with self-reported SCI to examine whether the same set of motor speech features improved estimation of episodic memory, executive function, and working memory scores.

## Methods

### Participant recruitment and eligibility

A cross-sectional community sample of older adults was recruited from the Island Study Linking Aging and Neurodegenerative Disease (ISLAND), for which a full protocol has been previously published [27]. ISLAND was launched in Tasmania, Australia, in 2019 as a 10-year public health initiative to educate residents aged 50 years and over on reducing their modifiable dementia risk factors [27].

ISLAND participants were invited to complete the TAS Test protocol, an online battery of speech and motor-cognitive tests (full protocol previously published) [2]. In brief, TAS Test comprises five sections, each focusing on different tests and abilities: (1) video hand movement and FT tests, (2) keyboard tapping tests, (3) visuomotor tests for visuoperceptual deficits and reaction times, (4) visuospatial tests of ability and working memory, and (5) tests of motor speech (DDK) and language abilities (verbal picture description).

The participants who elected to be involved self-administered TAS Test remotely and without researcher supervision at their convenience between October and December 2022 by logging into the TAS Test website through a personalized link, providing consent, and following the on-screen text and audiovisual instructions. If participants needed to resume testing after an accidental exit or interruption, they could log in again through their personal link and resume from their most recent incomplete test. Participants were not able to navigate backwards to retake completed test items.

#### HC and SCI group criteria

Because this study targeted adults without overt symptoms, we excluded any participants who reported conditions associated with cognitive or movement difficulties. Those who responded “yes” to any of the following ISLAND background survey questions were excluded: “Have you been told by a doctor that you have dementia?”; “Have you been told by your doctor that you have a memory impairment, but they were uncertain if you have dementia?”; “Have you been diagnosed with delirium?”; and “Have you been diagnosed with a central nervous system degenerative disease, e.g. Parkinson’s, Huntington’s, Multiple Sclerosis?” TAS Test survey questions were also considered, and participants were excluded if they reported any prior diagnoses of “Parkinson’s disease,” “Multiple Sclerosis,” or “Mild Cognitive Impairment” in these items.

**Fig. 1:**
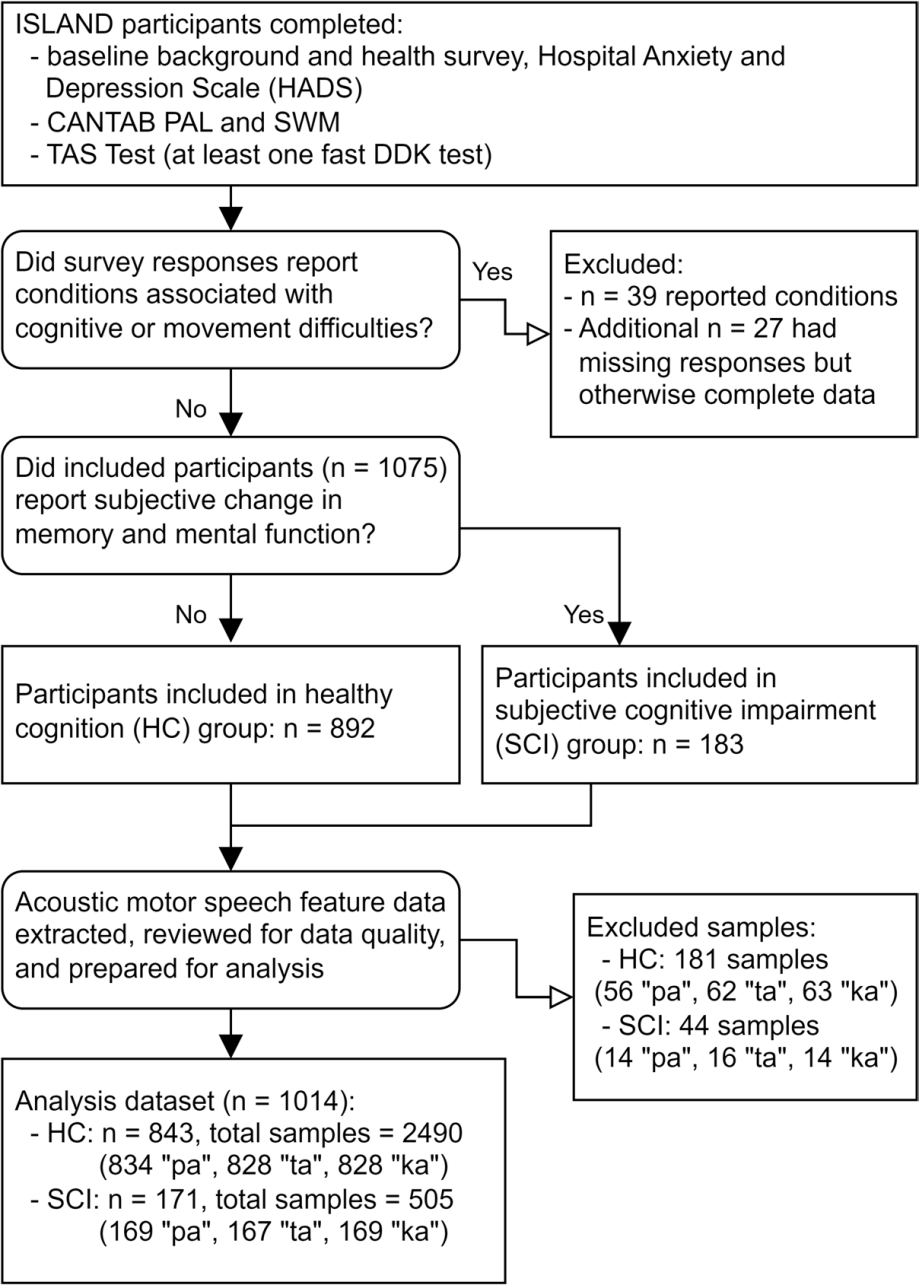
Flow diagram of participant recruitment, cognitive group classification criteria, and motor speech samples included in the final analysis dataset

Following this screening, participants were assigned to our HC or SCI groups depending on their response to the ISLAND background survey question, “Have you noticed a substantial change in your memory and mental function in recent years?” Participants who responded “no” were assigned to the HC group; those who responded “yes” were assigned to the SCI group. Figure 1 illustrates the participant recruitment and classification process.

### Demographic and clinical information

Participants’ demographic and clinical details were surveyed at the time of their recruitment to ISLAND (between 2019 and 2020). From these baseline data, we included the following details in our models: age in years (at the median TAS Test login date), sex (self-report response options: male, female, other, prefer not to say), highest level of education attained, anxiety and depression scores as reported using the Hospital Anxiety and Depression Scale (HADS), and “yes” or “no” responses to the question “Have you been diagnosed with a psychiatric disorder e.g. depression, psychosis, bipolar disorder, anxiety disorder?” The HADS and psychiatric items were included to adjust for effects of anxiety and depression on test performance and were not applied as exclusion criteria. Further characteristics of the ISLAND cohort are available in the protocol and interim results [27, 28].

### Cognitive assessment

ISLAND participants remotely completed two tests selected from the Cambridge Neuropsychological Test Automated Battery (CANTAB) [29] in August 2021.

#### Paired associates learning (PAL)

The PAL test assesses visual episodic memory through the memorization and location of increasing numbers of hidden patterns concealed in boxes on-screen. The test typically takes 8 min to administer. The score used in this study is the PALTEA6 score, which represents participants’ total errors on the six-pattern sequence, adjusted for incomplete or failed trials.

#### Spatial working memory (SWM)

The SWM test suite assesses working memory and executive function (planning and decision-making). Participants memorize and identify the locations of hidden pattern sequences and search for predetermined numbers of hidden tokens. The test typically takes 15 min to administer.

The scores used in this study are SWMBE6 for spatial working memory and SWMS for executive function. SWMBE6 measures the number of times during a six-token search trial that a participant revisits a box in which they had previously found a token. SWMS measures search strategy through the number of unique boxes a participant uses to commence their search processes over the course of all their trials.

### Motor speech data collection

Within the motor speech section of the TAS Test protocol, participants completed three fast, 10-s monosyllabic DDK tests (“pa", “ta", and “ka”) in fixed order. Participants were presented with the following instructions in text and video, with the video including a demonstration by a speech pathologist (“pa” example described): “Please repeat the sound ‘pa’ as fast as you can, like this: ‘pa-pa-pa…’. Keep going until you see the ‘Well done’ sign.” Once ready, participants commenced a 5-s countdown to each test by clicking a button labelled “Start the Test.” Fig. 1 in the Online Resource shows the steps of the “pa” test.

No standardized equipment or physical configuration of recording space was used, as participants were instructed to use their personal desktop or laptop computers to access the web-based TAS Test battery at home.

### Acoustic feature extraction pipeline

The fast “pa", “ta", and “ka” speech recordings were stored in single-channel WAV format at a variety of sample rates from 8 to 48 kHz since capture parameters varied depending on participants’ computer hardware and settings. All recordings with 8 kHz sample rates were excluded upon review due to distorted quality (three total; one “pa,” one “ta,” and one “ka”). Recordings of negligible size or length (likely due to issues during capture or upload) and those containing no utterances were also excluded. We applied non-stationary noise reduction using the Python package “noisereduce” (v3.0.2) [30] to reduce the influence of ambient noises.

**Fig. 2:**
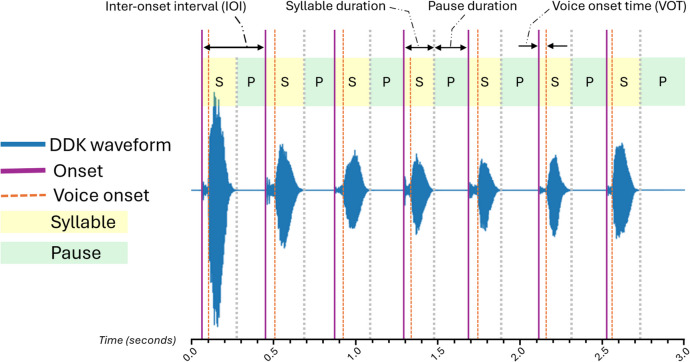
Annotated amplitude view adapted from a DDK recording showing seven syllable repetitions. The onset events mark the commencement of the syllable in each repetition; the inter-onset intervals (IOIs) are the time between sequential syllable onsets (i.e., time to complete each repetition cycle). The “pa", “ta", and “ka” syllables all begin with voiceless plosives (“p", “t", and “k”) and the voice onset events mark when the production of the voiced vowel (“a”) begins in each repetition. In this study, voice onset time (VOT) is measured as the duration between the speech onset event and the voice onset event. Each repetition is comprised of two zones: “syllable” during active speech production and “pause” for the segment between the end of the utterance and the start of the next repetition

For feature extraction, we used two methods to create a combined set of complementary features from the same set of preprocessed recordings. The first method was our custom Python script, which used the package “librosa” (v0.10.1) [31] to load recordings, normalize signals, evaluate relative changes in energy over time, and extract onset event times, and the “signal” submodule of “SciPy” (v1.13.0) [32] to locate local waveform peaks. Individual features were then computed using our directly specified formulae. The second method was developed by Redenlab® and has been previously published [33–35]. To facilitate the description of our full feature set, Fig. 2 annotates key events and classifications on a waveform excerpt adapted from a DDK recording.

As Fig. 2 shows, the main events and zones in the DDK tests are the onset of speech in each repetition, voice onset (vibration of the vocal folds) at the commencement of the vowel sound, and the distinction between the spoken “syllable” and silent “pause” zones within each repetition. Table 1 presents our full list of features, grouped by their underlying qualities to reflect the key events or zones used in their calculation. Features derived from the qualities of “onset,” “IOI,” and “energy” were extracted using our custom script, and those derived from the qualities “syllable,” “pause,” and “voice onset” were extracted using the methods developed by Redenlab®.

**Table 1:** Motor speech feature definitions grouped by underlying quality

Quality	Feature name	Definition^a^
Onset	Onset count	Total number of syllable onsets
	Onset rate	Total syllable onset count divided by total sample duration (per second)
Inter-onset interval	IOI mean	Average inter-syllable onset interval (IOI) (seconds)
	IOI SD	Standard deviation (SD) of IOI (seconds)
	Inverse min. IOI	Inverse of minimum IOI (per second)
	Decrement on IOI	Logarithmic ratio derived from first and last IOIs, normalized by total count of IOIs
	Inverse IOI mean	Mean of inverse IOI (per second)
	Inverse IOI COV	Coefficient of variation^b^ (COV) of inverse IOI
Syllable	Articulation rate	Total syllable count divided by total speech duration (per second)
	Syllable duration mean	Average syllable duration (seconds)
	Syllable duration COV	Coefficient of variation of syllable duration
	Speech percent	Total speech duration as a percentage of total sample duration
	Speech to pause ratio	Ratio of total speech duration to total pause duration
Pause	Pause mean	Average pause duration (seconds)
	Pause COV	Coefficient of variation of pause duration
	Pause percent	Total pause duration as a percentage of total sample duration
Voice onset	VOT mean	Average voice onset time^c^ (VOT) (seconds)
	VOT COV	Coefficient of variation of VOT
Energy	Onset strength COV	Coefficient of variation of normalized onset strengths (relative changes in energy over time)

^a^Units provided where applicable

^b^Coefficient of variation (COV) = standard deviation/mean

^c^See Fig. 2 for VOT detail

### Statistical analysis

The goal of this analysis was to determine whether any of the DDK features improved cognitive score model fit over demographic and clinical variables, rather than exploring individual feature significance and relationships. To this end, regression models were fitted to 18 combinations of factors from the two cognitive groups (HC or SCI), three cognitive score outcomes (PALTEA6 [episodic memory], SWMS [executive function], and SWMBE6 [working memory]), and three DDK tests. Null model variables were participant age, sex, highest level of education, presence of a psychiatric disorder diagnosis, and baseline HADS anxiety and depression scores. In R, cognitive scores were modelled using generalized linear models with outcome distributions suitable for count data: Poisson for executive function (“glm” function, base R v4.4.0), and negative binomial for episodic and working memory to account for overdispersion (“glm.nb” function, “MASS” v7.3–60.2) [36]. The “dredge” function of the “MuMIn” package (v1.47.5) [37] was used to evaluate whether DDK features made significant contributions to model fit over the null variables in any of the 18 test cases. This was achieved by using “dredge” to fit, evaluate, and rank models in each case, starting with the fixed set of null variables, followed by the null variables plus every linear combination of at least one feature. No interaction terms were included. To reduce computational cost and pairwise collinearity between DDK features, we prohibited the simultaneous inclusion of DDK features in any single model if their pairwise correlation coefficient exceeded 0.6 in all three DDK tests.

All model selection was performed using the corrected Akaike Information Criterion (AICc) [38]. Models containing DDK features were deemed to explain significantly more variance than null models if ΔAICc (computed as AICc_null_ − AICc_speech_) exceeded the rule of thumb threshold of two [39]. In each test case, if at least one model containing at least one feature was found to be superior to the null model, this was interpreted as supporting evidence that the DDK test improved estimation of the applicable cognitive outcome. Nagelkerke’s pseudo-*R*^2^ (*R*^2^_N_) is reported as an estimate of variance explained.

As a supplemental analysis into whether motor speech features improved prediction of HC vs. SCI, we fitted logistic regression models to each syllable-based subset of our data (i.e., “pa", “ta", and “ka”). Using the R package “pROC” [40], we compared receiver operating characteristic (ROC) curves for models fitted with null variables only to models fitted with all null model variables plus all motor speech features.

## Results

### Participants

We included data from 1014 participants without missing values in their eligibility, demographic, clinical, or cognitive variables, and for whom features from at least one DDK recording were available. Twenty-seven participants were excluded due to incomplete survey data. HC group criteria were met by 843 participants, and 171 participants met criteria for the SCI group. Summary details for the HC, SCI, and excluded participant groups are shown in Table 2.

**Table 2:** Summary of participant demographics and clinical details, cognitive scores, and DDK recordings

Measure	HC group (*n* = 843)	SCI group (*n* = 171)	Excluded^a^ (*n* = 27)
Age			
Mean (SD)	66.9 (7.3)	66.3 (7.3)	68.0 (7.0)
Median (min, max)	67 (51, 92)	66 (52, 82)	68 (54, 79)
Gender (%total)			
Female	614 (72.8%)	121 (70.8%)	21 (77.8%)
Male	229 (27.2%)	50 (29.2%)	6 (22.2%)
Highest level of education (% total)			
High school	83 (9.8%)	13 (7.6%)	4 (14.8%)
Certificate or apprenticeship (including cert 2, 3, or 4)	58 (6.9%)	25 (14.6%)	1 (3.7%)
Diploma/associate degree	170 (20.2%)	30 (17.5%)	5 (18.5%)
Bachelor’s degree	194 (23.0%)	39 (22.8%)	5 (18.5%)
Higher university degree (honors, graduate, diploma, masters, or PhD)	338 (40.1%)	64 (37.4%)	11 (40.7%)
Other	0 (0%)	0 (0%)	1 (3.7%)
Psychiatric disorder diagnosis (% total)			
No	715 (84.8%)	119 (69.6%)	20 (74.1%)
Yes	128 (15.2%)	52 (30.4%)	7 (25.9%)
Baseline HADS anxiety score^b^			
Mean (SD)	5.0 (3.4)	6.5 (3.9)	5.3 (3.6)
Median (min, max)	5 (0, 18)	6 (0, 18)	5 (0, 14)
Baseline HADS depression score^b^			
Mean (SD)	2.5 (2.2)	4.7 (3.2)	4.2 (2.5)
Median (min, max)	2 (0, 13)	4 (0, 14)	4 (0, 11)
Hearing impairment (% total)^c^			
No	701 (83.2%)	140 (81.9%)	17 (63.0%)
Yes	141 (16.7%)	31 (18.1%)	10 (37.0%)
Corrected hearing impairment (% of those with hearing impairment)^c^			
No	65 (46.1%)	16 (51.6%)	8 (80.0%)
Yes	74 (52.5%)	15 (48.4%)	2 (20.0%)
Wear corrective glasses (% total)^c^			
No	100 (11.9%)	11 (6.4%)	4 (14.8%)
Yes	740 (87.8%)	160 (93.6%)	23 (85.2%)
Episodic memory: PALTEA6^d^			
Mean (SD)	4.1 (4.4)	4.5 (4.9)	4.2 (5.9)
Median (min, max)	3 (0, 21)	3 (0, 22)	2 (0, 20)
Executive function: SWMS^d^			
Mean (SD)	7.7 (2.8)	7.9 (2.6)	8.1 (2.9)
Median (min, max)	8 (2, 13)	8 (2, 12)	8 (3, 13)
Working memory: SWMBE6^d^			
Mean (SD)	3.2 (3.3)	3.4 (3.4)	4.2 (3.7)
Median (min, max)	2 (0, 14)	3 (0, 12)	5 (0, 11)
Number of DDK recordings			
“pa”	834	169	27
“ta”	828	167	27
“ka”	828	169	25

^a^Participants ineligible due to incomplete personal data

^b^HADS anxiety and depression scores: normal, 0–7; borderline abnormal, 8–10; abnormal, 11–21. Participants with abnormal scores were offered support service information and contact details

^c^Missing responses: hearing impairment (*n* = 1 HC), corrected hearing impairment (*n* = 2 HC), wear corrective glasses (*n* = 3 HC)

^d^CANTAB scores: For all three of the selected tests, a lower score represents better performance. The minimum possible score for both PALTEA6 and SWMBE6 is zero; for SWMS, one

The number of DDK recordings included in each group varied across test types due to the results of data preprocessing and feature extraction. The counts reported in Table 2 were the totals remaining after preparing the data for analysis. From the HC group, 56 “pa” samples (6% of the total) were excluded, as were 62 “ta” samples (7%), and 63 “ka” samples (7%). From the SCI group, 14 “pa” samples (8%), 16 “ta” samples (9%), and 14 “ka” samples (8%) were excluded. Issues included variable speaking volumes which fell too quiet for microphones and differences in home internet connections, hardware, or configurations affecting file upload or sound quality.

**Fig. 3:**
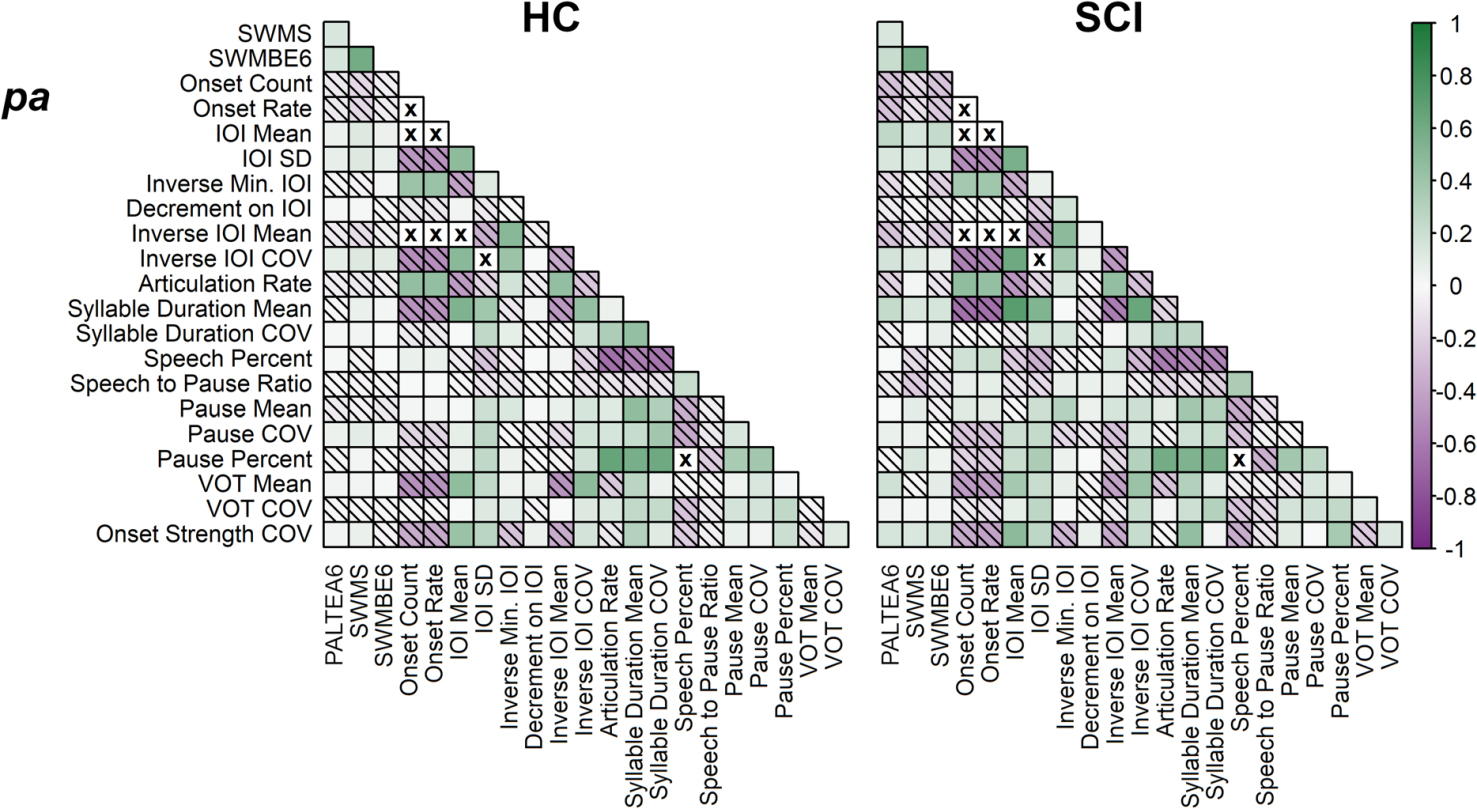
Pairwise correlation coefficients between cognitive scores (PALTEA6 [visual episodic memory], SWMS [executive function], and SWMBE6 [spatial working memory]) and all motor speech features listed in Table 1, measured from “pa” tests (“ta” and “ka” results are shown in Online Resource Fig. 2). Correlations are displayed separately for each participant cognitive group (healthy cognition [HC] or subjective cognitive impairment [SCI]). The feature pairs excluded from simultaneous selection in modelling are indicated by “x” (correlations not shown). Diagonal hatching indicates negative correlations

Figure 3 shows the pairwise correlation coefficients of all motor speech features (measured from “pa” tests) and cognitive scores. The correlations from the “ta” and “ka” tests are included in Online Resource Fig. 2. The cells marked with crosses indicate the feature pairs which exceeded the correlation threshold described in “Methods” and were excluded from simultaneous selection in models.

### Stronger associations with executive function in HC participants

The results for both participant groups are displayed in Table 3. In both the HC group and the SCI group, the addition of motor speech features achieved significant improvements in fit (i.e., ΔAICc > 2) over null models for all three cognitive outcomes.

**Table 3:** Results in model significance (ΔAICc > 2), fit (*R*^2^_N_), and proportion of additional variance explained (Δ*R*^2^_N_) by participant group, cognitive domain, and DDK test

Participant group	Cognitive domain (test score)	DDK test	ΔAICc^a^	*R*^2^_N, null_ (%)	*R*^2^_N, best_^b^ (%)	Δ*R*^2^_N_ (%)
Healthy cognition (HC)	Episodic memory (PALTEA6)	*“pa”*	*5.8*	*9.0*	*10.1*	*1.1*
		*“ta”*	*2.1*	*8.6*	*9.3*	*0.7*
		“ka”	0.0	8.5	8.5	0.0
	Executive function (SWMS)	*“pa”*	*6.2*	*11.9*	*13.0*	*1.1*
		*“ta”*	*2.7*	*11.7*	*12.2*	*0.5*
		*“ka”*	*7.1*	*11.9*	*13.5*	*1.6*
	Working memory (SWMBE6)	*“pa”*	*2.5*	*6.4*	*7.4*	*1.0*
		“ta”	0.0	6.3	6.5	0.2
		“ka”	0.1	5.9	6.2	0.3
Subjective cognitive impairment (SCI)	Episodic memory (PALTEA6)	*“pa”*	*7.4*	*4.1*	*9.5*	*5.4*
		“ta”	1.3	3.9	6.0	2.1
		*“ka”*	*7.1*	*4.2*	*13.1*	*8.9*
	Executive function (SWMS)	*“pa”*	*3.8*	*13.6*	*16.7*	*3.1*
		“ta”	0.0	13.8	13.8	0.0
		“ka”	0.0	13.6	13.6	0.0
	Working memory (SWMBE6)	*“pa”*	*4.5*	*8.3*	*16.9*	*8.6*
		“ta”	0.0	7.8	7.8	0.0
		“ka”	0.0	7.5	7.5	0.0

^a^Italicized rows indicate the cases in which a model with at least one motor speech feature achieved a significantly improved fit (ΔAICc > 2) over the demographic and clinical variable-only (null) model (AICc_null_). ΔAICc = AICc_null_ − AICc_best_

^b^The model deemed “best” in each case was the one which returned the lowest AICc (i.e., AICc_best_). This was either the null model or a model which included at least one motor speech feature

In the HC group, features from all three DDK tests improved models of executive function. The two strongest results found in the HC group were in this domain (“ka” ΔAICc = 7.1, followed by “pa” ΔAICc = 6.2). Significant improvements were also found in episodic memory (“pa” ΔAICc = 5.8 and “ta” ΔAICc = 2.1) and working memory (“pa” ΔAICc = 2.5).

Performance on the “ka” test was associated with executive function only. Features from the “pa” test were the only ones associated with improvements in all three cognitive domains.

### Stronger associations with episodic memory in participants with SCI

As Table 3 shows, a total of four significant associations were found in the SCI group. No cognitive domain was found to be associated with performance in all three DDK tests. The two strongest results were both in episodic memory (“pa” ΔAICc = 7.4 and “ka” ΔAICc = 7.1). The next best result was in working memory (“pa” ΔAICc = 4.5), followed by executive function (“pa” ΔAICc = 3.8). Performance on the “ta” test was not found to improve models in any of the cognitive domains. In parallel with the HC group, the “pa” test was the only one associated with improvements in each cognitive domain.

### Differences in feature prevalence

**Fig. 4:**
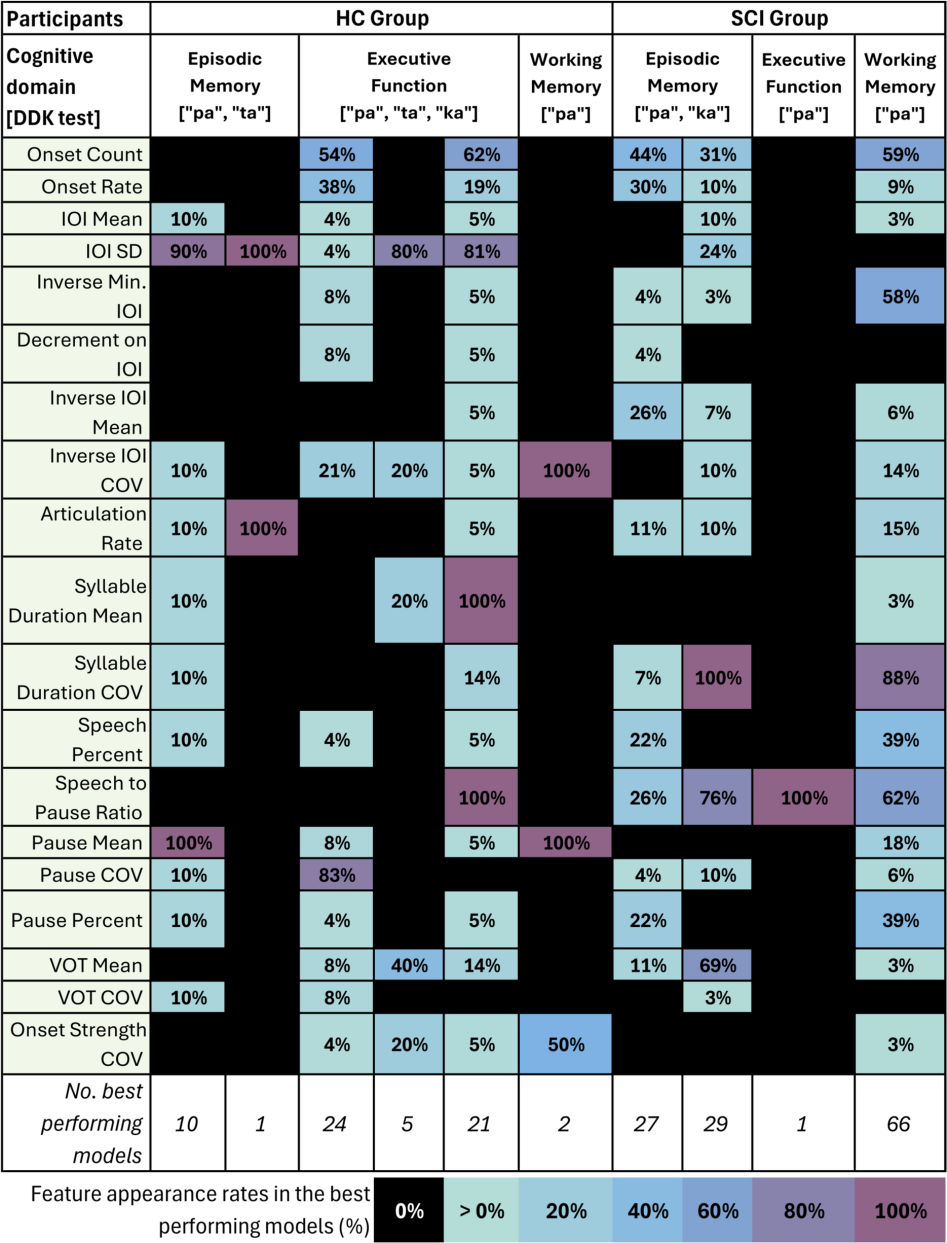
Summary of feature inclusion rates in the set of approximately equivalent best performing models for each participant group, cognitive domain, and DDK test case. In each case, the set of models considered to be these candidates for best fit were those which achieved a significant improvement over their respective null model and were ranked within ΔAICc < 2 of the model with the lowest AICc. A 100% appearance rate means that a feature was included in all the candidates for best performing model in that specific test case. Expanded feature definitions are available in Table 1

To help inform future investigations into specific aspects of motor speech performance, Fig. 4 provides a breakdown of individual feature prevalence in each significant test case. The models summarized are those which achieved significant improvements relative to null and were either the best-ranked model or approximately equivalent in fit (i.e., AICc within two units).

Across the full set of results, every feature appeared at least once. The top five features accounted for approximately 55% of all appearances and were syllable duration coefficient of variation (COV; 14.5%), speech to pause ratio (14.3%), onset count (13.4%), inverse of minimum IOI (6.7%), and IOI standard deviation (SD; 6.1%). The three features which appeared the least were onset strength COV, decrement on IOI, and VOT COV. We note this aggregation disproportionately represents the larger result sets such as working memory in the SCI group.

Comparing across participant groups instead, the feature IOI SD appeared across almost all significant test cases in the HC group, except for working memory and the “pa” test. In the SCI group, however, this feature was only associated with the test case for episodic memory and “ka". Conversely, the speech to pause ratio was associated with every significant case in the SCI group, but only with one case in the HC group (executive function and “ka”).

To generalize across “pa” cases, pause COV and the structurally related features speech percent and pause percent appeared in most cases for both participant groups. The inverse IOI mean showed associations with the SCI group but not HC.

Focusing on individual cognitive domains, articulation rate was associated with all episodic memory test cases. For executive function, results were concentrated in the HC group due to minimal results in the SCI group. The HC group cases shared associations with IOI SD, inverse IOI COV, VOT mean, and onset strength COV. The HC and SCI working memory cases both shared inverse IOI COV, pause mean, and onset strength COV.

The results of our supplementary analysis into the classification of HC vs. SCI groups are available in the Online Resource (as Table 1 and Figs. 3, 4, and 5). In brief, inclusion of “pa”- or “ta”-measured motor speech features significantly improved (*p* < 0.05) classification performance over models fitted using null variables only.

## Discussion

Performance on short motor speech tests improved estimation of cognitive performance in the domains of episodic memory, executive function, and working memory. These three tests summed to only 30 s of active speech time per participant and achieved superior results beyond predictions using demographic (age, sex, education) and psychological (depression and anxiety) information alone.

In older adult participants without any cognitive symptoms or prior diagnoses (the HC group, *n* = 843), performance on each of the three DDK tests improved estimation of scores in all three cognitive domains. This partially supported our first and second hypotheses. Our first hypothesis expected features from all three DDK tests would improve models of episodic memory in this group, but only features from the “pa” and “ta” tests showed this association. Further, only one “ta”-based model of borderline significance was found, which may be a consequence of searching over a large variable combination space in the modelling process. Our second hypothesis anticipated that features from all three DDK tests would improve models of executive function and working memory. While features from all three tests did demonstrate associations with executive function, only the “pa” test was found to be associated with working memory. Across the HC results, model fit improved the most in executive function using “ka” features (Δ*R*^2^_N_ = 1.6%, total variance explained 13.5%).

Our secondary analysis in the SCI group (*n* = 171) found similar patterns. In this group, performance features from the “pa” and “ka” tests were associated with episodic memory. In terms of model fit improvements, the “pa” features doubled, and “ka” features tripled, the proportions of variance explained. This is notable as both SCI and reductions in episodic memory are established proxy measures for AD risk. Features from the “pa” test alone were found to be associated with executive function and working memory. The best fitting model was in working memory using “pa” features (Δ*R*^2^_N_ = 8.6%, total variance explained 16.9%).

The full results suggest that associations between FT performance and episodic memory, executive function, and working memory in healthy older adults [18] extend to motor speech performance, and to participants with SCI. In FT, features from the dominant hand outperformed those from the nondominant hand in all three cognitive domains (with only dominant features significant in working memory) [18]. In this study, the “pa” test yielded results in all six combinations of cognitive domain and group, compared to two each for “ta” and “ka". The “pa” features performed the best across almost all results and were the only ones significant in working memory. This raises questions of how associations between cognitive functions and plosive motor recruitment vary by place of articulation (bilabial “p", alveolar “t", or velar “k”), and whether bilabial motor associations are more dominant.

Interest is developing in tongue strength as a possible predictor of cognitive decline since it is affected by sarcopenia, which is, itself, a risk factor [41]. As articulation of the “ta” and “ka” syllables involves tongue movement, tongue strength may influence their associations with cognitive decline. Including measures of tongue strength could offer a localized way to adjust DDK performance for oral frailty and to explore performance differences across different stages of cognitive decline. In our results, “pa” had the broadest range of associations, suggesting that tongue-independent articulation tasks may have wider screening applications, at least in the earliest stages of cognitive decline. Unfortunately, the fixed order of DDK tests in the TAS Test protocol (1, “pa”; 2, “ta”; 3, “ka”) may have influenced our results, along with fatigue, since DDK tests occurred after approximately 20 min of testing. Future work would benefit from randomizing or counter-balancing test order to validate syllable-specific associations.

Executive function has previously been linked with motor speech performance in younger adults [23, 24] and this study supports this association in older adults. Features from all three DDK tests improved estimation of executive function in the HC group, but only the “pa” test was associated with the SCI group (with only one candidate model identified). The only feature to appear in the SCI model was the speech to pause ratio. The same feature appeared only in the “ka” results for the HC group but in all candidate models. Feature prevalence varied over the HC cases, with the most frequent feature in “pa” models being pause COV. Both “pa” and “ka” shared moderate selection preference for onset-based features, and “ta” and “ka” tended toward IOI SD. The most notable difference between the HC and SCI groups was the overall decrease in results. Possible neural mechanisms for this require additional investigation. In healthy adults undertaking single-repetition DDK tasks, the areas activated in brain networks of motor control and phonological processing regions changed across age groups without a decrease in performance [42]. Our results require validation but may suggest that SCI is associated with a departure from the pattern of network reorganization expected in healthy aging.

A key strength of this study is that our models adjusted for important demographic and psychological covariates, which were set as the benchmark variables for estimating cognitive test performance. Other strengths include the breadth of investigation across cognitive conditions (i.e., asymptomatic HC group, subtle complaints in the SCI group), and the use of participant-collected data without researcher supervision or specialist equipment. We extracted a wide range of acoustic features from three established DDK tests, using complementary methods to capture subtle variations in motor speech performance.

It is important to note that the CANTAB tests studied relate to the specific cognitive domains of episodic memory, executive function, and working memory, and do not represent global cognitive function. Participants could also have had impairments in other domains which were not measured. We also note that changes in associations between motor speech performance and cognition over time cannot be inferred from this study due to its cross-sectional design. We reported associations with episodic memory and SCI, both of which are relevant to AD risk but not exclusive. These data suggest that longitudinal evidence of associations with cognitive decline (for instance, subtle changes in episodic memory, or later diagnoses of MCI and AD) will be valuable for finding and characterizing risk profiles specific to AD. A longitudinal investigation of these associations may be possible through ISLAND. We acknowledge, however, the limited cultural and linguistic diversity of participants recruited from ISLAND, most of whom were female, highly educated, and of White, Northern European ancestry. This limits our findings from generalizing to a broader domestic or international population.

Longitudinal and meta-analytical evidence supports the validity of using a single question to classify SCI in participants [43, 44]. However, the eligibility criteria used did not preclude the possibility that some participants classified as HC may have had presymptomatic neurodegenerative disease. Similarly, it is possible that some participants classified as SCI may have had clinically significant impairment which was undiagnosed at the time of survey completion. Participants in either group may also have had difficulty understanding the medical terminology used in the personal health surveys, incorrect responses to which could have contributed to group misclassification. Since participants were recruited from a highly educated cohort, we expect the risk of misunderstanding to have been low, but we are unable to confirm this as comprehension was not assessed.

The remote collection of speech samples introduced substantial variation between participants in hardware, test environment and its spatial configuration, and internet connection quality. This has likely affected results. Test delivery and data collection through smartphones, while also subject to variability, may have the potential to offer greater community reach and accessibility compared to desktop and laptop computers [45, 46]. While delivery of the test battery through smartphones may help accommodate participants with lower digital literacy, written survey items and instructions may remain a barrier to participants with lower literacy levels. Although the selected DDK tests were provided with text and audiovisual instructions to facilitate access, and were intended to impose minimal language- and culture-specific requirements, members ultimately required computer and internet access, digital literacy, and reading ability to participate. Further studies are also needed to account for the effects on motor speech performance of factors such as fluency in everyday speech, denture usage, and differences in hearing ability. These factors are relevant across all ages but will be especially important in distinguishing pathological and aging-associated changes in motor speech function. Hearing loss, for example, is a known risk factor for dementia and, although the risk can be mitigated with correction [4], a slim majority of our SCI group participants with hearing impairment reported no use of corrective devices. In adolescents, hearing impairment is associated with DDK performance [47], but further research is needed into these relationships and the effects of corrective device use in older adults.

The promising results of this study facilitate future work investigating the significance and relationships of specific motor speech features with cognition, as this type of interpretation is not supported by the current study design. Additional lines of investigation could include similar analyses using features which represent DDK accuracy, comparisons between self- and maximal-paced tests to represent motor reserve, and extensions into the clinically useful sequential trisyllabic “pataka” test. Further, investigation of associations between motor speech performance and blood-, CSF-, or imaging-based biomarkers of AD pathology may assist with linking at-risk cognitive functions to underlying AD pathology via non-invasive speech measures. Emerging multimodal and artificial intelligence-based methods for early detection of neurodegenerative diseases [48, 49] may offer alternative approaches to feature extraction, analysis, and pooling with other modes of testing such as FT.

In summary, this study demonstrates that performance on short, self-administered motor speech tests is associated with cognition in older adults. Performance features from the “pa” DDK test improved estimation of test scores in episodic memory, executive function, and working memory in both participant groups (HC and SCI). This study establishes the potential of motor speech tests to be part of the solution in the urgent search for non-invasive, cost-effective, and community-scalable screening tools for AD risk.

## Supplementary Information

Below is the link to the electronic supplementary material.

Supplementary file1 (PDF 896 KB)

## Data Availability

The authors may share the de-identified data with qualified investigators whose proposal of data use has been approved by an independent review committee.
