# Supplementary material for: Rapid repetitive syllable sounds associate with episodic memory, executive function, and working memory in cognitively healthy and subjectively impaired older adults
Source: GeroScience. 2025 Jun 24;47(6):6683–98. doi: 10.1007/s11357-025-01739-x (PMC12638598; doi:10.1007/s11357-025-01739-x)
Supplement: Supplementary file 1 — Supplementary file1 (PDF 896 KB) [file 11357_2025_1739_MOESM1_ESM.pdf]

Online Resource: Supplementary Information

"Rapid repetitive syllable sounds associate with episodic memory, executive function, and working memory in cognitively healthy and subjectively impaired older adults"

Contents **Page**

Contents	Page
FIGURE 1. Participant instructions and steps for DDK tests of motor speech ability	2
FIGURE 2. Pairwise correlation coefficients of motor speech features from "ta" and "ka" tests and cognitive scores	3
FIGURE 3. Receiver Operating Characteristic (ROC) curve and Area Under Curve (AUC) for motor speech features measured from the "pa" DDK test	4
FIGURE 4. ROC curve and AUC for motor speech features measured from the "ta" DDK test	5
FIGURE 5. ROC curve and AUC for motor speech features measured from the "ka" DDK test	6
TABLE 1. Tests for differences in HC vs. SCI logistic model classification accuracy with the addition of motor speech features over null demographic and clinical variables	7

FIGURE 1. Participant instructions and steps for DDK tests of motor speech ability

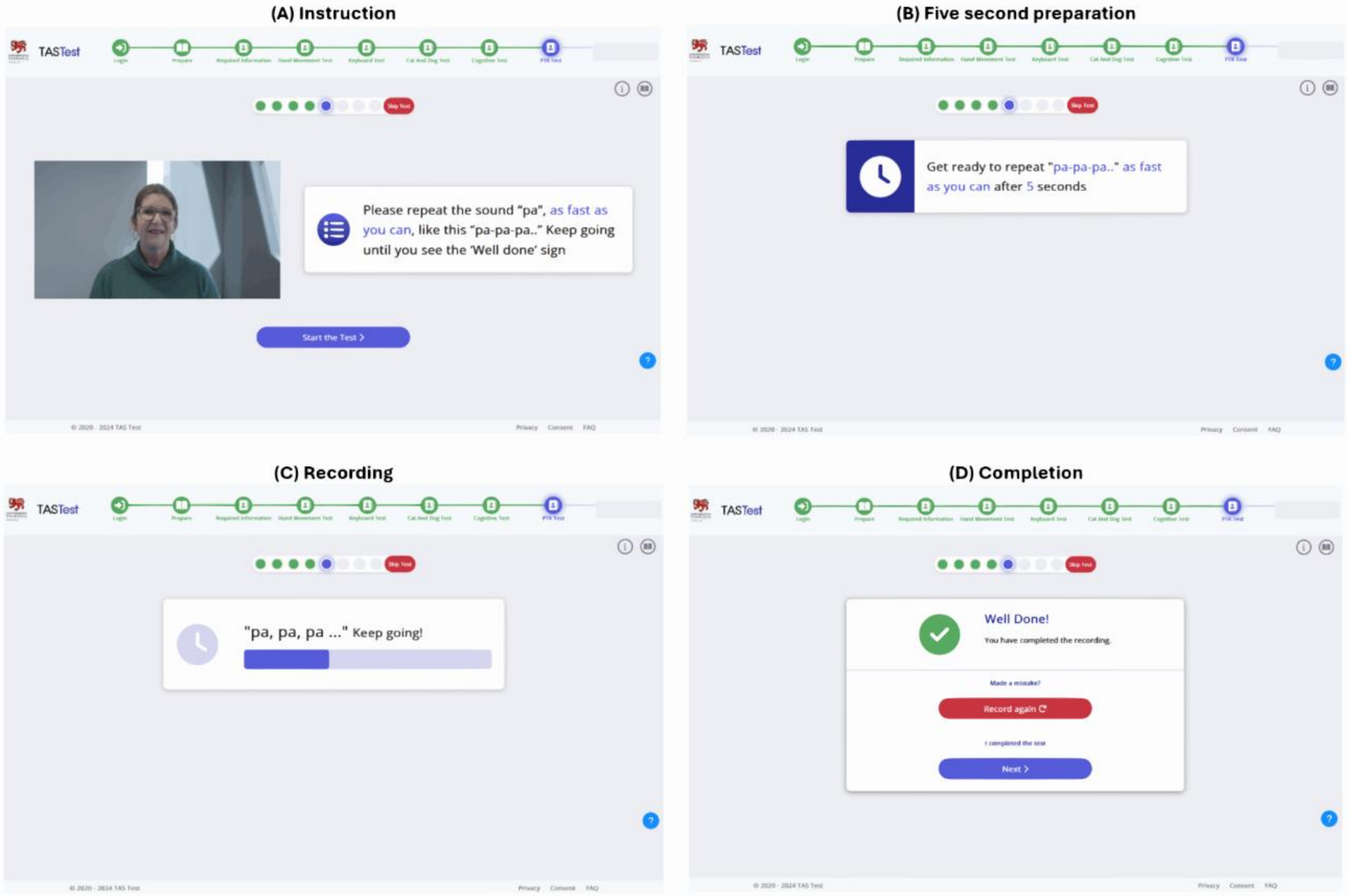

Figure 1 caption: The steps of the fast, monosyllabic DDK tests in the TAS Test battery (illustrated for the "pa" condition). Panel (A): Participants are shown text and looped audiovisual instructions including a short demonstration. Participants choose when to click "Start the Test". Panel (B): Participants are shown a reminder of the instruction while an automatic 5 second countdown displays the time until the recording begins. Panel (C): While participants attempt the test, which is automatically limited to 10 seconds (the duration is not disclosed in the instructions), the display shows an instruction prompt and a progress bar for the length of time remaining. Panel (D): After the timer concludes, the "Well Done!" screen is displayed. Participants have the option to retake the test if they made a mistake (redirection to screen (B), three attempts maximum) or to continue to the next item in the battery.

FIGURE 2. Pairwise correlation coefficients of motor speech features from "ta" and "ka" tests and cognitive scores

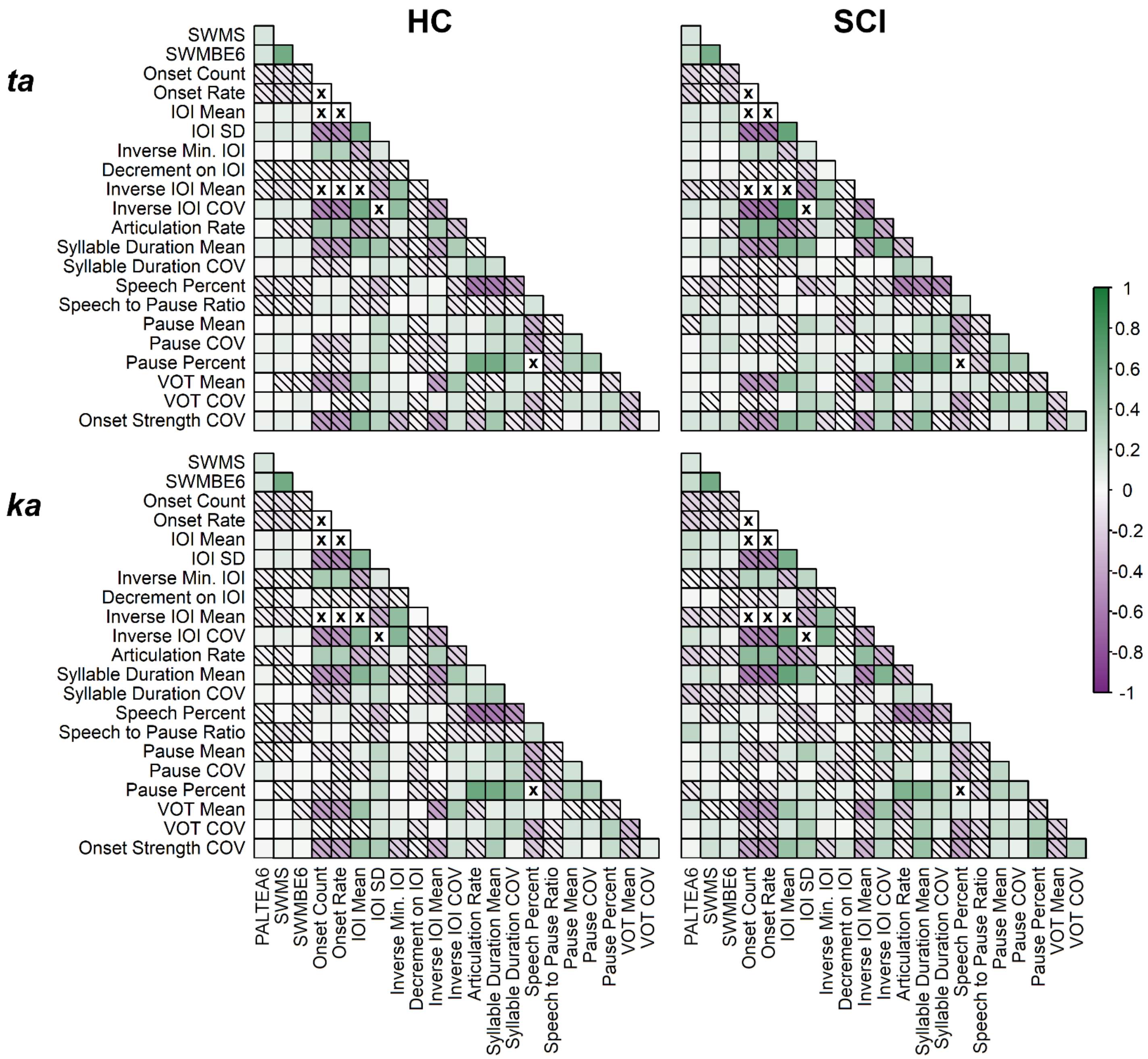

Figure 2 caption: Pairwise correlation coefficients between cognitive scores (PALTEA6 [visual episodic memory], SWMS [executive function], and SWMBE6 [spatial working memory]) and all motor speech features listed in Table 1, measured from "ta" and "ka" tests. Correlations are displayed, broken down by participant cognitive group (healthy cognition [HC] or subjective cognitive impairment [SCI]) and DDK test syllable ("ta", or "ka"). The feature pairs excluded from simultaneous selection in modelling are indicated by "x" (correlations not shown). Diagonal hatching indicates negative correlations.

FIGURE 3. Receiver Operating Characteristic (ROC) curve and Area Under Curve (AUC) for motor speech features measured from the “pa” DDK test

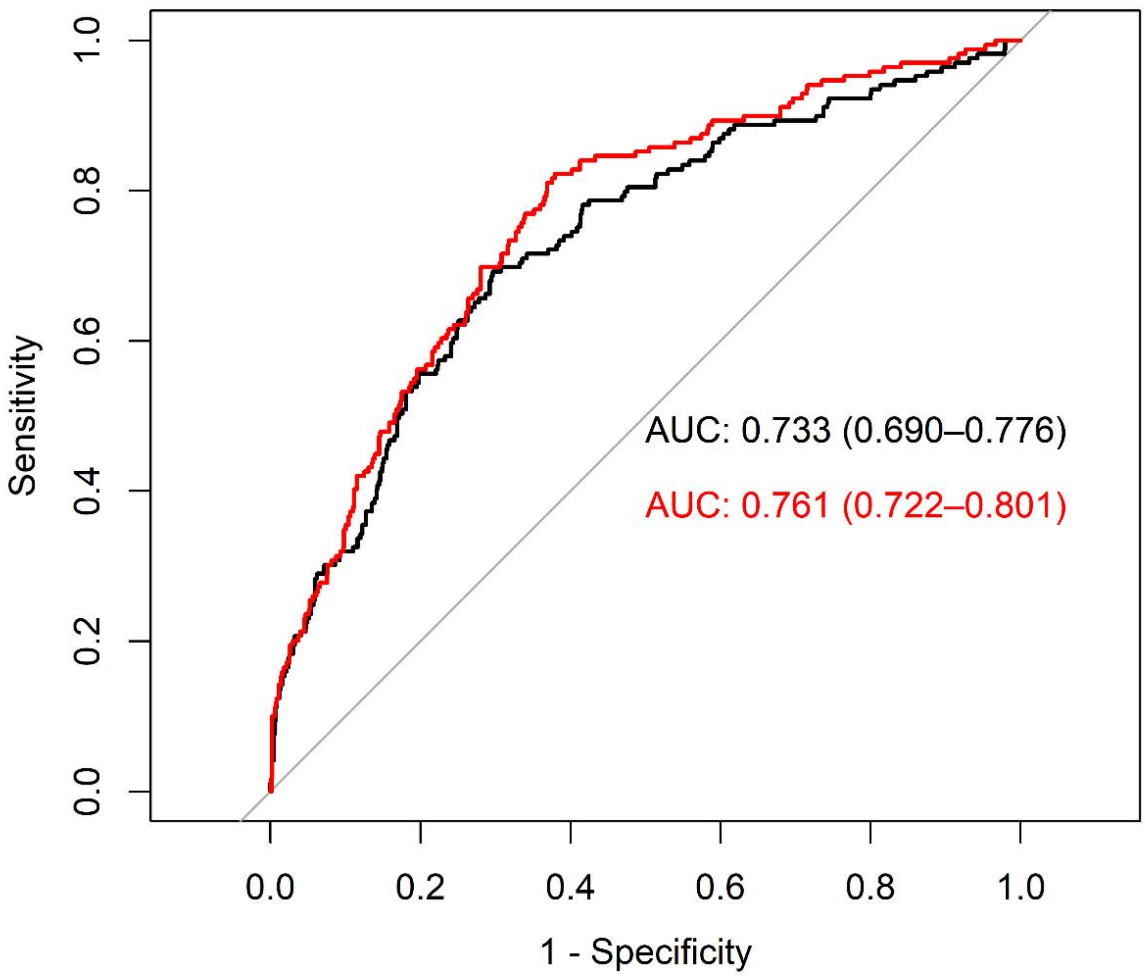

Figure 3 caption: Receiver Operating Characteristic (ROC) curves and 95% CIs for Area Under Curve (AUC) for SCI classification models using data from the “pa”-based DDK tests. The black ROC curve (and the topmost AUC estimate) shows the classification performance of the null model (fitted using only the demographic and clinical variables specified in Methods). The red curve shows the performance of the classification model fitted with all null variables plus all motor speech features. As shown in Online Resource Table 1, this improvement (larger AUC when motor speech features were included) was found to be significant.

FIGURE 4. ROC curve and AUC for motor speech features measured from the "ta" DDK test

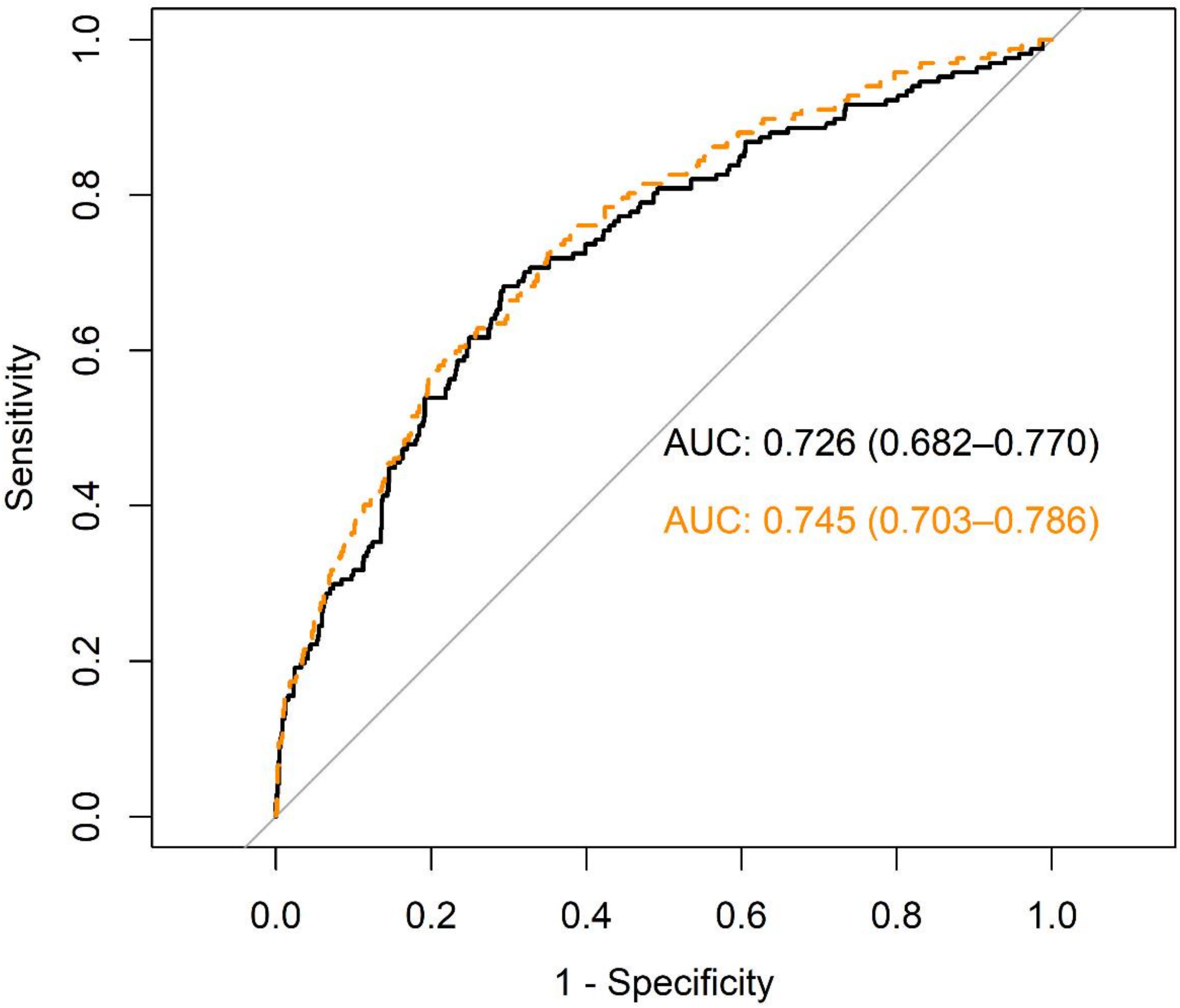

Figure 4 caption: Receiver Operating Characteristic (ROC) curves and 95% CIs for Area Under Curve (AUC) for SCI classification models using data from the "ta"-based DDK tests. The black ROC curve (and the topmost AUC estimate) shows the classification performance of the null model (fitted using only the demographic and clinical variables specified in Methods). The orange dashed curve shows the performance of the classification model fitted with all null variables plus all motor speech features. As shown in Online Resource Table 1, this improvement (larger AUC when motor speech features were included) was found to be significant.

FIGURE 5. ROC curve and AUC for motor speech features measured from the “ka” DDK test

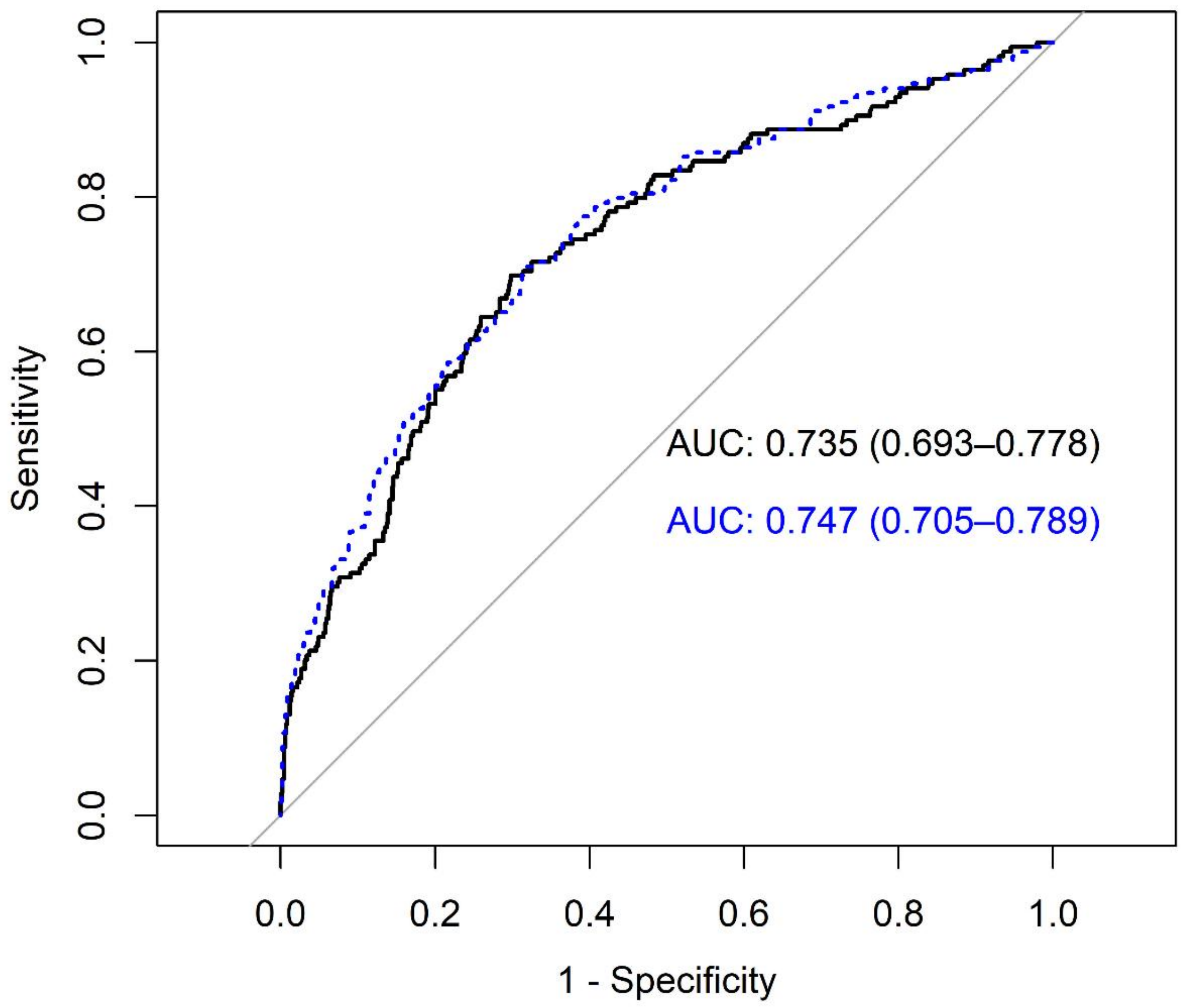

Figure 5 caption: Receiver Operating Characteristic (ROC) curves and 95% CIs for Area Under Curve (AUC) for SCI classification models using data from the “ka”-based DDK tests. The black ROC curve (and the topmost AUC estimate) shows the classification performance of the null model (fitted using only the demographic and clinical variables specified in Methods). The blue dotted curve shows the performance of the classification model fitted with all null variables plus all motor speech features. As shown in Online Resource Table 1, the addition of motor speech features to the null model in this case did not result in a significant improvement in HC vs. SCI classification accuracy.

TABLE 1. Tests for differences in HC vs. SCI logistic model classification accuracy with the addition of motor speech features over null demographic and clinical variables

DDK test syllable	Area under Receiver Operating Characteristic (ROC) curve (AUC) (95% CI)			DeLong's test for two correlated ROC curves[c]	
	n	Null model[a]	Motor speech feature model[b]	Z	p-value
"pa"	1003	0.733 (0.690 – 0.776)	0.761 (0.722 – 0.801)	-2.5423	0.011
"ta"	995	0.726 (0.682 – 0.770)	0.745 (0.703 – 0.786)	-1.9659	0.049
"ka"	997	0.735 (0.693 – 0.778)	0.747 (0.705 – 0.789)	-1.1808	0.238

[a] Null models fitted with demographic and clinical variables only.

[b] Motor speech feature models fitted with all null model variables plus all motor speech features.

[c] Conducted in R using roc.test() from the package 'pROC' to test for differences in AUCs of paired ROC curves.
